# Size-Dependent Strength and Reliability of Resin Composite Blocks and Nanoceramics for Computer-Aided Design/Computer-Aided Manufacturing (CAD/CAM) Restorations

**DOI:** 10.3390/ma19122564

**Published:** 2026-06-13

**Authors:** Fernando Ledesma-Renedo, Eva Paz, Francisco Martínez-Rus, Miguel Ángel Rodríguez-Pérez, Guillermo Pradíes

**Affiliations:** 1 Analysis of Techniques, Material and Instruments Applied to Digital Dentistry and CAD/CAM Procedures Research Group, Department of Conservative and Prosthetic Dentistry, Faculty of Odontology, Complutense University of Madrid, 28040 Madrid, Spain; ferledes@ucm.es (F.L.-R.); gpradies@ucm.es (G.P.); 2 Institute for Research in Technology (IIT), Mechanical Engineering Department, ICAI School of Engineering, Comillas Pontifical University of Madrid, 28015 Madrid, Spain; eva.paz@iit.comillas.edu; 3 Cellular Materials Laboratory (CellMat), Condensed Matter Physics Department, Campus Miguel Delibes Science Faculty, University of Valladolid, 47011 Valladolid, Spain; marrod@fmc.uva.es

**Keywords:** dental materials, ceramics, composite resins, computer-aided design, flexural strength

## Abstract

Background: Mechanical reliability and size-dependent strength behavior remain critical concerns for CAD/CAM restorative materials. This study evaluated resin-based CAD/CAM materials, including resin composite blocks (RCBs) and nanoceramics. The influence of specimen size on flexural strength and the applicability of Weibull-based strength predictions were assessed by comparing experimental and Weibull-predicted values. Methods: Twelve CAD/CAM materials were investigated, including ten resin-based materials and two controls (lithium disilicate ceramic and polymethyl methacrylate). Rectangular specimens (1 × 4 × 14 mm and 1 × 12 × 14 mm) were tested using a three-point bending test. Flexural strength, modulus, and resilience were calculated. Reliability and size dependence were assessed using two-parameter Weibull statistics and effective-volume-based predictions. Data were analyzed using statistical tests selected according to data distribution characteristics (α = 0.05). Results: RCBs exhibited higher flexural strength, modulus, and resilience than nanoceramics (*p* < 0.05). Weibull analysis indicated higher reliability and limited size dependence for RCBs, whereas nanoceramics showed greater variability. The ceramic control exhibited the expected reduction in strength with increasing specimen size. In contrast, resin-based materials showed inconsistent responses to changes in specimen size. Prediction error analysis revealed variable agreement between predicted and experimental values, indicating that agreement with classical Weibull assumptions was material-dependent. Conclusions: Resin-based CAD/CAM materials demonstrated limited size-dependent behavior compared with brittle ceramics. The reduced agreement between experimental and Weibull-predicted values suggests that effective-volume scaling may have limited applicability for these contemporary materials and should be interpreted cautiously on a material-specific basis.

## 1. Introduction

Computer-aided design/computer-aided manufacturing (CAD/CAM) restorative materials have become widely used in prosthodontics due to their ability to provide standardized industrial manufacturing, improved mechanical properties, and simplified clinical workflows [1,2,3]. Among these materials, resin-based CAD/CAM blocks have gained increasing attention because they combine a polymer matrix with reinforcing ceramic fillers, aiming to integrate favorable characteristics of both ceramics and polymers [4,5,6].

Several categories of resin-based CAD/CAM materials are currently available, including resin composite blocks (RCBs) and nanoceramics [7,8,9]. In the present study, resin-based CAD/CAM materials were used as the overarching term, whereas RCBs and nanoceramics were defined as specific subgroups and referred to consistently throughout the manuscript. These materials are manufactured under controlled industrial conditions, which allow a higher degree of polymerization and optimized filler distribution compared with conventional direct composites [10,11]. As a result, they have been proposed for a wide range of indirect restorations, including inlays, onlays, veneers, full-coverage crowns, and implant-supported restorations [12,13,14].

The mechanical behavior of these materials has been widely investigated, particularly with respect to flexural strength, elastic modulus, and fracture resistance [15,16,17]. Flexural strength is considered one of the most clinically relevant mechanical parameters because restorative materials are frequently subjected to complex bending stresses during mastication [18]. Three-point bending tests are therefore commonly used to evaluate the mechanical performance of CAD/CAM materials [19]. However, conventional mechanical testing primarily provides information regarding strength values and does not necessarily explain material reliability or the influence of defect populations on fracture behavior, particularly under different loading configurations or stressed volumes.

From a clinical and materials science perspective, understanding strength reliability is particularly relevant because restorations of different dimensions and geometries may experience different stress distributions and effective stressed volumes during function. Although CAD/CAM materials are used in restorations ranging from thin veneers to full-coverage crowns and implant-supported restorations, the influence of specimen dimensions and stressed volume on their mechanical behavior remains insufficiently understood. Furthermore, changes in stressed volume may affect the probability of encountering critical defects, thereby influencing strength variability and fracture behavior. In this context, evaluating the relationship between stressed volume and strength variability may provide additional insight into the mechanisms governing fracture behavior in contemporary resin-based materials.

In brittle materials such as dental ceramics, strength variability is typically explained by flaw-controlled fracture mechanics. According to the weakest-link theory, the probability of failure depends on the distribution of internal defects, and larger stressed volumes are more likely to contain critical flaws [20,21]. This behavior leads to the so-called size effect, in which larger specimens tend to exhibit lower measured strength. Weibull statistics are commonly used to describe this phenomenon and to evaluate the reliability of brittle materials [22,23,24].

However, the applicability of classical Weibull-based scaling models to contemporary resin-based CAD/CAM materials remains unclear. The heterogeneous microstructure of these materials may alter the relationship between flaw population and strength distribution, potentially affecting the expected size dependence observed in conventional ceramics [25,26,27]. Despite their increasing clinical use, previous investigations of resin-based CAD/CAM materials have primarily focused on conventional mechanical properties, including flexural strength, elastic modulus, fracture resistance, and wear behavior [15,16,17,18,19]. Although these studies have contributed substantially to the understanding of their basic mechanical performance, important aspects related to strength reliability and failure behavior remain insufficiently explored.

In particular, the influence of specimen dimensions on strength variability and the applicability of classical Weibull-based approaches to these contemporary materials have not been adequately investigated. Therefore, the present study extends current knowledge by integrating conventional mechanical characterization with reliability analysis and experimental validation of Weibull effective-volume predictions, providing additional insight into the size-dependent behavior of resin-based CAD/CAM materials.

Therefore, the aim of this study was to evaluate the mechanical reliability and size-dependent strength behavior of resin composite blocks and nanoceramics used for CAD/CAM restorations and to investigate whether classical Weibull-based scaling assumptions adequately describe their mechanical response. Furthermore, experimentally measured strength values were compared with theoretical Weibull predictions to assess the applicability of effective-volume scaling in these materials, thereby evaluating the validity of classical Weibull assumptions for contemporary resin-based materials.

The null hypothesis was that neither the material subgroup nor specimen geometry would significantly influence the mechanical behavior of the CAD/CAM materials evaluated, and that the experimental results would be consistent with classical Weibull-based strength predictions.

## 2. Materials and Methods

This study evaluated ten resin-based CAD/CAM materials with similar clinical indications. A lithium disilicate glass-ceramic (IPS e.max CAD; EM, Ivoclar Vivadent, Schaan, Liechtenstein) and a polymethyl methacrylate-based polymer (G-Cam; GC, Graphenano Nanotechnologies, Valencia, Spain) were included as ceramic and polymer control materials, respectively. Material specifications, classifications, manufacturer-reported compositions, and batch numbers are presented in Table 1.

For each of the twelve materials, thirty rectangular specimens were prepared in two geometries: fifteen 4 mm wide specimens (1 × 4 × 14 mm) and fifteen 12 mm wide specimens (1 × 12 × 14 mm). In accordance with ISO 6872:2024 [16], CAD/CAM blocks were sectioned under water cooling using a high-speed precision cutting machine (IsoMet 1000; Buehler, Lake Bluff, IL, USA) equipped with diamond wafering blades. An IsoMet 30HC blade (Buehler) was used for Brava (BR), Grandio-Block (GD), Shofu Block (SB), Cerasmart 270 (CS), Brilliant Crios (BC), Tetric CAD (TC), Lava Ultimate (LU), Experimental (EX), Estellite P-block (ES), and GC. An IsoMet 15LC blade (Buehler) was used for Katana Avencia (KA) and EM due to their higher hardness.

The 12 mm specimens were obtained by transverse sectioning of the CAD/CAM blocks. The 4 mm specimens were subsequently prepared from the 12 mm specimens by additional cuts performed at 4.7 mm intervals, generating oversized strips. This oversizing was intentionally used to compensate for material loss associated with the cutting blade thickness (approximately 0.5 mm) and subsequent grinding and polishing procedures. The remaining material from each 12 mm section was discarded. Final specimen dimensions were achieved by water-cooled grinding and polishing using silicon carbide abrasive papers (P280, P400, P800, and P1200; CarbiMet, Buehler). Subsequently, all specimens were chamfered using P2500 silicon carbide paper (CarbiMet, Buehler). EM specimens were crystallized following the manufacturer’s firing protocol (10 min at 850 °C, heating rate 30 °C/min; Programat P500, Ivoclar Vivadent). Specimen dimensions were verified at three locations using a digital micrometer (Mitutoyo, Tokyo, Japan). Specimens exhibiting visible chipping or dimensional deviations greater than ±50 µm were excluded. Materials were coded (A–L) to allow blinded testing.

Specimen allocation was randomized using Microsoft Excel (Microsoft Corporation, Redmond, WA, USA) to minimize potential variability related to material heterogeneity. All specimens were tested immediately after preparation at room temperature (21 ± 1 °C).

**Figure 1:**
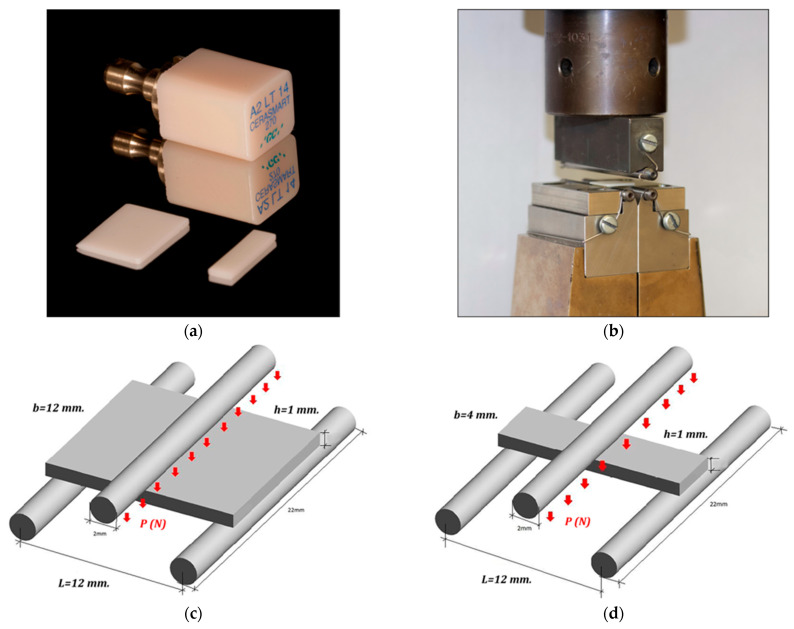
Specimen preparation and three-point bending test configuration. (**a**) Representative CAD/CAM block (Cerasmart 270) and specimens corresponding to the two geometries evaluated: 12 mm wide specimen (left) and 4 mm wide specimen (right). (**b**) Photograph of the three-point bending fixture used during mechanical testing. (**c**) Schematic representation of the 4 mm specimen geometry (14 × 4 × 1 mm) and loading configuration. (**d**) Schematic representation of the 12 mm specimen geometry (14 × 12 × 1 mm) and loading configuration. Red arrows indicate the direction of load application during the three-point bending test.

Mechanical testing was performed using three-point bending under uniaxial flexural loading using the configuration shown in Figure 1. All tests were conducted with a universal testing machine (Instron 3369; Instron, Norwood, MA, USA) equipped with a calibrated 1-kN load cell. The same testing assembly was used for both specimen geometries and consisted of two cylindrical support rollers and a cylindrical loading roller that were free to rotate during testing, with a support span of 12 mm. Specimens were centered beneath the loading roller, and a crosshead speed of 0.5 mm/min was applied and controlled using Bluehill 3 software (Instron) until failure.

Force (N) and displacement (mm) data were continuously recorded to generate stress–strain curves. From these curves, the main flexural properties were determined based on standard beam theory. Flexural strength (*σ_f_*), defined as the maximum flexural stress at failure (MPa), was calculated as:

(1) $$\sigma_{f}=\frac{3PL}{2bh^{2}}$$

Flexural modulus (*Ε_f_*), defined as the slope of the linear elastic region of the stress–strain curve obtained during the flexural test (GPa), was calculated as:

(2) $$E_{f}=\frac{L^{3}S}{4bh^{3}}$$

The resilience modulus (*U_r_*), defined as the elastic strain energy per unit volume stored up to fracture (MPa), was calculated as:

(3) $$U_{r}=\frac{\sigma_{f}^{2}}{2E_{f}},$$

where *P* is the fracture load (N), *L* the support span length (mm), *b* the specimen width (mm), *h* the specimen thickness (mm), and *S* the slope of the linear portion of the load–displacement curve (N/mm).

Representative scanning electron microscopy (SEM) images were obtained for all evaluated materials to qualitatively assess filler morphology and microstructural organization. In addition, fractographic analysis was performed on representative fractured specimens from selected resin composite blocks (GD and TC) and nanoceramics (CS and LU) to identify fracture origins and characterize local fracture mechanisms. SEM analysis was performed using a scanning electron microscope (FlexSEM 1000; Hitachi, Taito-ku, Japan). Specimens were prepared by sectioning CAD/CAM blocks (Isomet 1000, Buehler), polishing them with carbide abrasive paper (P180; CarbiMet, Buehler), and fracturing them after immersion in liquid nitrogen for 5 min to preserve their internal structure. Fractured surfaces were gold sputter-coated for 40 s at 30 mA and 5 × 10^−2^ mbar prior to imaging. Observation parameters, including kV settings, magnification, detector type, and image scale, are indicated in the corresponding micrographs. Fractographic observations were used qualitatively to evaluate crack propagation patterns, filler debonding, particle pull-out, crack deflection, and other local fracture features.

Statistical analyses were performed using SPSS software (version 22.0; SPSS Inc., Chicago, IL, USA), with the significance level set at α = 0.05. Normality of the data was assessed using Shapiro–Wilk and Kolmogorov–Smirnov tests. As most datasets did not meet normality assumptions, nonparametric methods were selected for intergroup comparisons among materials. Accordingly, intergroup comparisons were performed using the Kruskal–Wallis test followed by Mann–Whitney U tests. Comparisons between specimen geometries (4 mm vs. 12 mm) within each material were performed using analysis of variance (ANOVA), as these datasets met the assumptions of normality. This approach ensured that the statistical analyses were selected according to the characteristics and distribution of the data.

Flexural strength data were further analyzed using a two-parameter Weibull distribution. The cumulative probability of failure (*P_f_*) was expressed as:

(4) $$P_{f}=1-exp\left[-{\left(\frac{\sigma }{\sigma_{\theta }}\right)}^{m}\right],$$

where *σ* is the applied flexural stress; *σ_θ_* is the characteristic flexural strength, defined as the stress corresponding to a cumulative probability of failure of 63.2% according to the Weibull distribution function; and *m* is the Weibull modulus, which describes the scatter of strength data. Higher *m* values indicate lower variability and greater strength reliability.

The Weibull modulus (*m*) and characteristic strength (*σ_θ_*), together with their 95% confidence intervals, were determined from linear regression of Weibull-transformed data, where ln[−ln(1 − *P_f_*)] was plotted against ln(σ). In this representation, the slope of the fitted regression line corresponds to *m*, whereas *σ_θ_* was calculated from the intercept. Standard errors (SEs) were calculated from the residual sum of squares (RSS) of the fitted regression line.

To evaluate the influence of specimen geometry, the size effect was assessed using the effective-volume (*Ve*) concept derived from Weibull weakest-link theory. In contrast to the geometric volume of a specimen, the effective volume represents the portion of material effectively subjected to the applied tensile stress field and therefore contributing to the probability of failure. Because the tensile stress distribution in three-point bending is non-uniform, only a fraction of the specimen volume is exposed to the highest stress levels; consequently, the effective volume is smaller than the actual geometric volume. This parameter accounts for the non-uniform stress distribution inherent to flexural loading and constitutes the basis of classical Weibull size-effect analysis.

For rectangular specimens tested under three-point bending, the effective volume was calculated as:

(5) $$Ve=\frac{Lbh}{\left\lfloor 2{\left(m+1\right)}^{2}\right\rfloor },$$

where *L*, *b*, and *h* represent the specimen length, width, and thickness, respectively, and *m* is the Weibull modulus obtained from the corresponding Weibull regression analysis.

The relationship between characteristic strength and effective volume was expressed as:

(6) $$\frac{\sigma_{1}}{\sigma_{2}}={\left(\frac{{Ve}_{2}}{{Ve}_{1}}\right)}^{\frac{1}{m}},$$

where *σ_1_* and *σ_2_* represent the characteristic strengths corresponding to specimen geometries 1 and 2, respectively, and *Ve_1_* and *Ve_2_* their corresponding effective volumes.

For each material, the 4 mm specimen geometry was used as the reference dataset. The corresponding Weibull parameters (*σ_θ_* and *m*) were introduced into Equation (6) to predict the characteristic strength expected for the 12 mm specimen geometry based on the effective-volume ratio. Predicted values were subsequently compared with the experimentally measured characteristic strengths obtained from the 12 mm specimens to assess the agreement with classical Weibull effective-volume scaling.

The prediction error (%) between predicted and experimental characteristic strength values was calculated as:

(7) $$Prediction\;error\;\left(\%\right)=\;\frac{\left[\sigma \;pred\;-\;\sigma \;exp\right]}{\sigma \;exp}\;\times \;100,$$

where σ pred is the predicted characteristic strength and σ exp is the experimentally measured characteristic strength.

## 3. Results

### 3.1. Morphological Characterization

**Figure 2:**
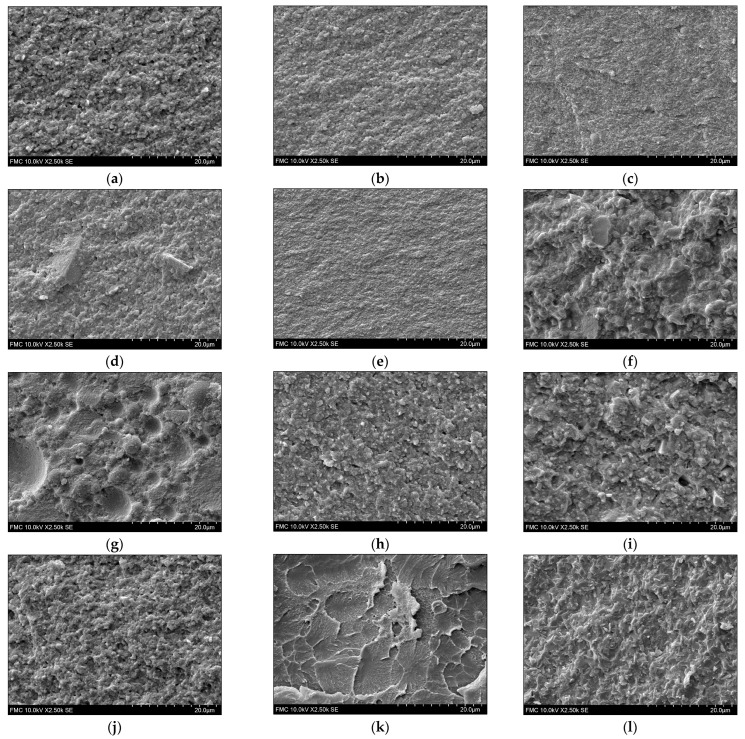
Representative SEM images of the evaluated materials: (**a**) Brava (BR), (**b**) Cerasmart 270 (CS), (**c**) Estellite P-block (ES), (**d**) Experimental (EX), (**e**) Katana Avencia (KA), (**f**) Lava Ultimate (LU), (**g**) Shofu Block (SB), (**h**) Brilliant Crios (BC), (**i**) Grandio-Block (GD), (**j**) Tetric CAD (TC), (**k**) G-Cam (GC), and (**l**) IPS e.max CAD (EM). Scale bars and imaging parameters are indicated in each micrograph.

Representative SEM images of the evaluated materials are shown in Figure 2. SEM analysis revealed differences in particle morphology and microstructural arrangement among the investigated materials. Resin composite blocks generally exhibited relatively homogeneous structural patterns with micro- to nanoscale particle distributions. Greater variability was observed among nanoceramics, with differences in particle size, filler organization, and the presence of structural heterogeneities. EX exhibited larger discrete particles and a more heterogeneous appearance, whereas ES showed agglomerated nano- and submicron fillers. SB presented spherical voids, while KA, CS, and LU exhibited more homogeneous particle distributions with minimal apparent defects. The polymer control (GC) displayed a smooth matrix morphology, whereas the ceramic control (EM) showed a dense glass-like structure. Overall, the evaluated materials demonstrated heterogeneous microstructural characteristics.

### 3.2. Mechanical Properties

**Figure 3:**
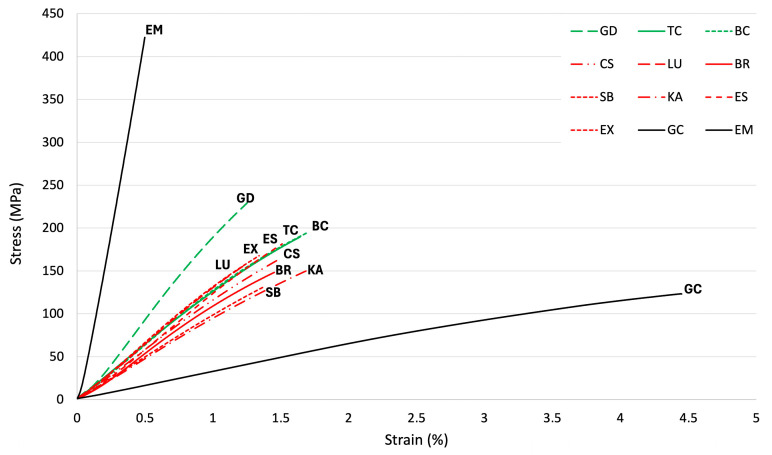
Stress–strain curves for the 4 mm specimen geometry.

**Figure 4:**
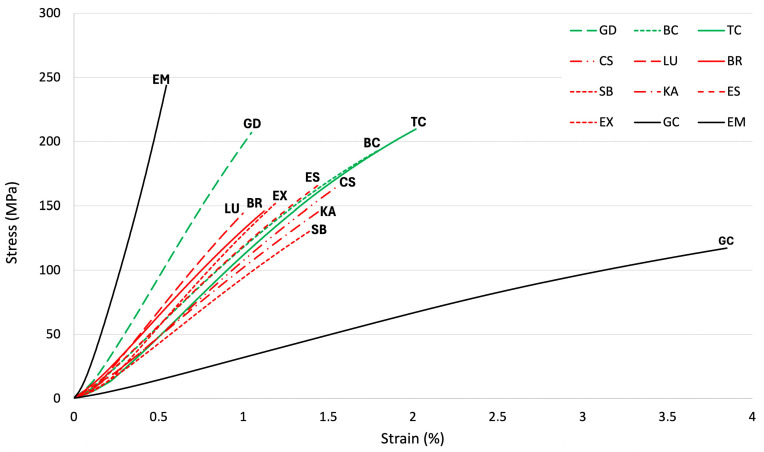
Stress–strain curves for the 12 mm specimen geometry.

Representative stress–strain curves for all materials and specimen geometries are shown in Figure 3 and Figure 4. Resin-based CAD/CAM materials exhibited predominantly brittle behavior in both configurations, with minimal or no plastic deformation. The control materials (gray curves) represented opposite mechanical responses, with the ceramic control (EM) exhibiting the steepest stress–strain slope and lowest strain values at failure, whereas the polymer control (GC) showed the lowest slope and the highest strain values. Resin-based materials exhibited an intermediate response between these two controls. Among them, resin composite blocks (green curves) generally showed steeper stress–strain slopes and higher stress values than nanoceramics (red curves) at comparable strain levels, consistent with their higher flexural strength and elastic modulus values summarized in Table 2.

Among the resin-based CAD/CAM materials, resin composite blocks (GD, TC, and BC) consistently showed higher flexural strength than nanoceramics in both specimen geometries (Table 2). The ceramic control exhibited the highest values (406.31 ± 62.50 MPa for 4 mm specimens), whereas the polymer control showed the lowest values (117.94 ± 7.53 MPa for 12 mm specimens). Variations related to specimen geometry were material-dependent, with some materials (e.g., TC and CS) showing higher strength in the 12 mm configuration, while others showed similar or lower values, indicating that no consistent size-dependent pattern was observed across the resin-based CAD/CAM materials evaluated.

Flexural modulus values showed a trend similar to that observed for flexural strength. All resin-based CAD/CAM materials exhibited substantially lower stiffness than the ceramic control (94.09 ± 7.12 GPa) and higher stiffness than the polymer control (3.46 ± 0.15 GPa). Among the resin-based CAD/CAM materials, GD exhibited the highest modulus values for both specimen geometries, whereas nanoceramics generally showed lower modulus values (Table 2).

Resilience modulus values further highlighted differences between material groups. RCBs generally exhibited higher resilience values than nanoceramics, whereas the polymer control showed the highest resilience overall (2.34 ± 0.33 MPa). Within individual materials, differences between specimen geometries were generally small, with limited variation between the 4 mm and 12 mm configurations (Table 2).

**Figure 5:**
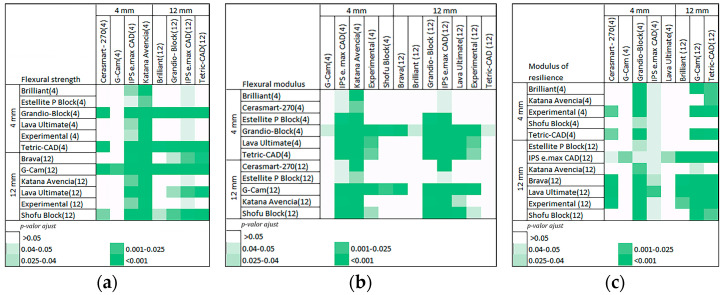
Graphical summary of statistically significant pairwise comparisons for mechanical properties among materials and specimen geometries: (**a**) flexural strength; (**b**) flexural modulus; (**c**) resilience modulus. Color maps represent adjusted *p* values obtained from post hoc Mann–Whitney U tests following Kruskal–Wallis analysis. Color intensity reflects the level of statistical significance, as indicated in the legend (*p* > 0.05 to *p* < 0.001).

Intergroup comparisons revealed statistically significant differences among the evaluated materials for all mechanical properties (flexural strength, flexural modulus, and resilience modulus). The Kruskal–Wallis test, followed by Mann–Whitney U post hoc analysis, identified statistically significant differences across multiple pairwise comparisons (*p* < 0.05 to *p* < 0.001). A graphical summary of the statistically significant pairwise comparisons is presented in Figure 5, where increasing color intensity reflects higher levels of statistical significance between groups.

Regarding the effect of specimen geometry, analysis of variance (ANOVA) revealed statistically significant differences between 4 mm and 12 mm specimens for several materials and mechanical parameters (Table 3). These differences were material-dependent, confirming that specimen geometry influenced the measured mechanical response without a consistent trend across all materials.

### 3.3. Weibull Analysis

Weibull parameters describing strength reliability are summarized in Table 4A,B. Weibull moduli (*m*) ranged from approximately 4.32 to 28.90, with higher values indicating greater reliability. Resin composite blocks exhibited consistently higher characteristic strength (*σ_θ_*) values than nanoceramics for both specimen geometries. Characteristic strength values followed a similar trend to flexural strength, with higher *σ_θ_* values generally observed for RCBs than for nanoceramics across both configurations.

**Figure 6:**
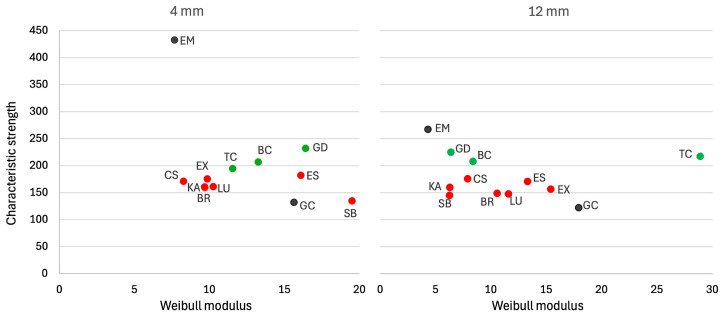
Relationship between Weibull modulus and characteristic strength for 4 mm and 12 mm specimens, comparing control materials (gray), resin composite blocks (green), and nanoceramics (red). For the 4 mm specimens, KA and BR exhibited nearly identical Weibull modulus and characteristic strength values and are therefore represented by a single overlapping point.

The relationship between Weibull modulus and characteristic strength is illustrated in Figure 6. Weibull modulus values varied among materials; however, TC exhibited a marked increase in *m* for the 12 mm specimens (11.55 to 28.90), indicating improved strength reliability in this configuration.

**Figure 7:**
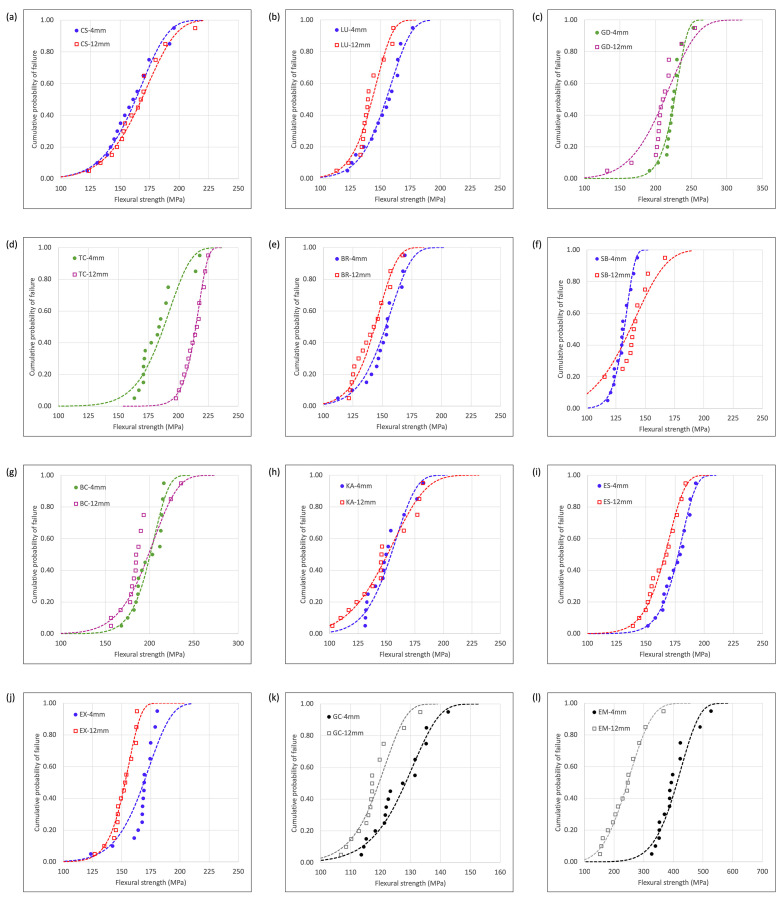
Weibull cumulative failure probability plots for both specimen geometries (4 mm and 12 mm) across all materials: (**a**) CS, (**b**) LU, (**c**) GD, (**d**) TC, (**e**) BR, (**f**) SB, (**g**) BC, (**h**) KA, (**i**) ES, (**j**) EX, (**k**) GC, and (**l**) EM. Control materials are shown in black/gray (GC, EM), resin composite blocks in green/purple (GD, TC, BC), and nanoceramics in blue/red (CS, LU, BR, SB, KA, ES, EX).

Weibull plots illustrating cumulative failure probability are presented in Figure 7 and demonstrate heterogeneous behavior among materials. Classical size-effect behavior was clearly observed in the ceramic control, with a reduction in *σ_θ_* from 432.11 MPa (4 mm) to 267.43 MPa (12 mm) and non-overlapping confidence intervals. The absence of overlap between confidence intervals supports the presence of a pronounced size effect in this material. In contrast, most resin-based CAD/CAM materials exhibited overlapping confidence intervals between specimen geometries, suggesting limited or inconsistent size-dependent behavior. In some cases (e.g., TC), *σ_θ_* increased from 194.05 MPa to 217.27 MPa with increasing specimen size.

The prediction error (%) between predicted and experimental characteristic strength values varied substantially among materials (Table 5). Lower prediction errors were observed for LU, GD, BR, ES, EX, and GC, indicating good agreement with classical size-effect predictions. In contrast, greater discrepancies were found in several resin-based materials and particularly in the ceramic control, reflecting deviations from ideal brittle behavior and suggesting a limited applicability of classical Weibull-based scaling for these materials.

### 3.4. Fractographic Analysis

**Figure 8:**
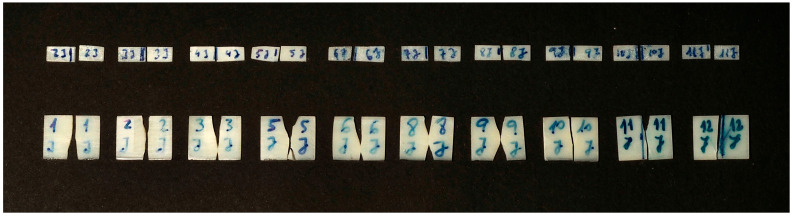
Representative fractured specimens of the nanoceramic Experimental (EX) material after three-point bending testing.

**Figure 9:**
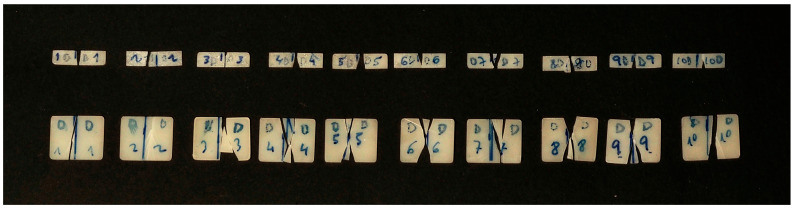
Representative fractured specimens of the resin composite block Tetric CAD (TC) after three-point bending testing.

Representative photographs of fractured specimens after three-point bending testing are shown in Figure 8 and Figure 9. Complete fracture was observed in all specimens. Although both material groups exhibited predominantly brittle failure, differences in fracture morphology were visually apparent. The nanoceramic specimens (Figure 8) generally showed simpler fracture trajectories with separation into fewer fragments, whereas the resin composite block specimens (Figure 9) frequently exhibited more complex fracture patterns, including secondary crack paths and multiple fracture fragments. These macroscopic observations are consistent with the differences in fracture surface morphology subsequently identified by SEM fractographic analysis.

**Figure 10:**
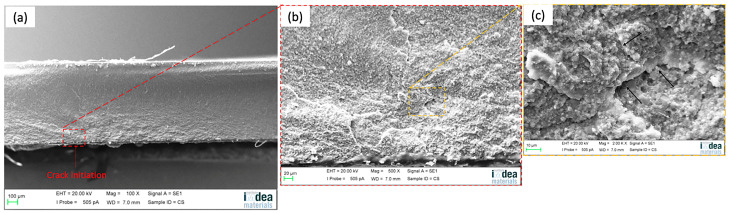
SEM fractographic analysis of a fractured flexural specimen of Cerasmart 270 (CS). (**a**) Low-magnification overview of the complete fracture surface obtained at ×100 magnification; (**b**) fracture surface obtained at ×500 magnification; (**c**) high-magnification detail at ×2000 magnification. Arrows indicate regions of interest associated with local fracture mechanisms events.

**Figure 11:**
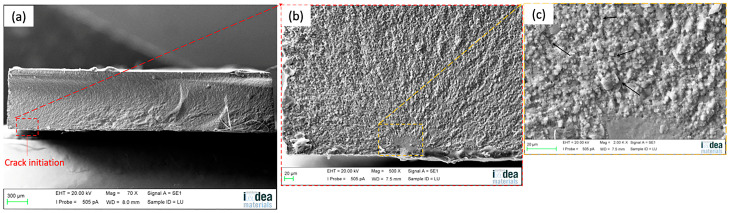
SEM fractographic analysis of a fractured flexural specimen of Lava Ultimate (LU). (**a**) Low-magnification overview of the complete fracture surface obtained at ×70 magnification; (**b**) fracture surface obtained at ×500 magnification; (**c**) high-magnification detail at ×2000 magnification. Arrows indicate regions of interest associated with local fracture mechanisms events.

**Figure 12:**
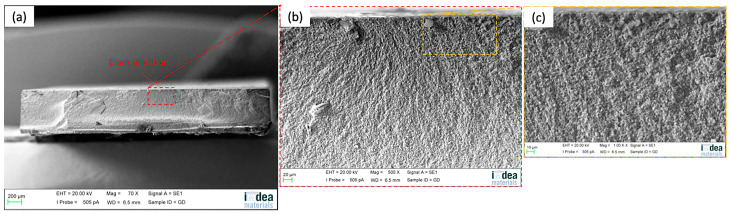
SEM fractographic analysis of a fractured flexural specimen of Grandio-Block (GD). (**a**) Low-magnification overview of the complete fracture surface obtained at ×70 magnification; (**b**) fracture surface obtained at ×500 magnification; (**c**) high-magnification detail at ×1000 magnification. Arrows indicate regions of interest associated with local fracture mechanisms events.

**Figure 13:**
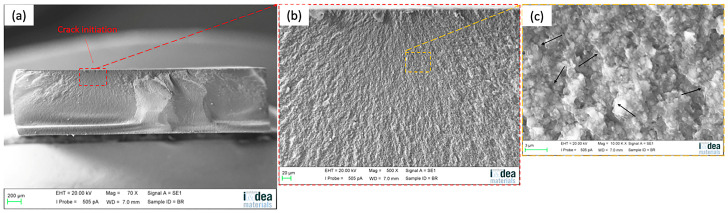
SEM fractographic analysis of a fractured flexural specimen of Tetric CAD (TC). (**a**) Low-magnification overview of the complete fracture surface obtained at ×70 magnification; (**b**) fracture surface obtained at ×500 magnification; (**c**) high-magnification detail at ×10,000 magnification. Arrows indicate regions of interest associated with local fracture mechanisms events.

Representative SEM fractographic analyses of selected nanoceramics (CS and LU) and resin composite blocks (GD and TC) are presented in Figure 10, Figure 11, Figure 12 and Figure 13. In all materials, the low-magnification images enabled visualization of the overall fracture topography and crack propagation patterns, whereas the higher-magnification images provided additional information regarding local fracture features and matrix–filler interactions.

The nanoceramics (CS and LU; Figure 10 and Figure 11) generally exhibited relatively smooth fracture surfaces with limited topographical variation. Filler debonding, particle pull-out, and localized interfacial defects were frequently observed, particularly at higher magnifications.

In contrast, the resin composite blocks (GD and TC; Figure 12 and Figure 13) exhibited rougher fracture surfaces characterized by fracture steps, secondary crack planes, and increased crack-path tortuosity. Evidence of crack deflection around filler particles and localized microcracking was also observed.

Overall, the fracture surfaces revealed differences in fracture morphology and crack propagation patterns between the two material subgroups.

## 4. Discussion

This study evaluated the mechanical behavior and reliability of resin-based CAD/CAM restorative materials, with particular emphasis on size-dependent strength and the applicability of Weibull-based scaling. Material type significantly influenced mechanical performance, whereas the effect of specimen geometry was material-dependent. Therefore, the null hypothesis was rejected.

Stress–strain analysis confirmed that resin-based CAD/CAM materials exhibit predominantly brittle behavior, with minimal macroscopic plastic deformation. However, a slight non-linearity in the nominally elastic region was consistently observed, in agreement with previous reports [18]. This behavior may be attributed to viscoelastic effects of the polymer matrix or to microstructural mechanisms such as interfacial debonding, rather than to true plasticity, suggesting a matrix-influenced fracture process [27]. In contrast, the ceramic control exhibited a linear elastic response up to fracture. Because the observed deviations from linearity were limited and occurred primarily near failure, the resilience modulus calculated using Equation (3) should be interpreted as a comparative estimate derived from linear elastic beam theory rather than as a direct measure of the total energy absorbed during fracture.

Resin-based CAD/CAM materials showed mechanical properties intermediate between those of the ceramic and polymer controls, consistent with previous studies [28,29]. This intermediate behavior reflects their heterogeneous microstructure and may contribute to stress distribution under functional loading conditions [1,15]. Within this group, resin composite blocks exhibited higher flexural strength and resilience than nanoceramics, indicating a greater capacity for elastic energy absorption prior to fracture [18,19,30,31,32,33,34,35]. These differences suggest that grouping both materials under a single category may mask relevant mechanical distinctions.

These distinctions may be related, at least in part, to differences in manufacturing techniques. Resin composite blocks are typically fabricated from highly filled resin matrices in which inorganic particles are dispersed within a polymerizable monomer system and subsequently cured under controlled temperature and pressure conditions, resulting in a relatively homogeneous composite structure [1,36]. In contrast, nanoceramics are manufactured using industrial processing strategies distinct from those used for resin composite blocks [4,10,31,37,38], resulting in differences in filler distribution, matrix–filler interactions, and microstructural organization.

The SEM observations supported the presence of microstructural differences among the evaluated materials. Resin composite blocks generally exhibited more homogeneous particle distributions, whereas nanoceramics showed greater variability in particle morphology and organization. These features may partially explain the differences observed in mechanical performance and reliability behavior. However, because the SEM analysis was qualitative and no quantitative image analysis was performed, these observations should be interpreted with caution.

Additional insight into the failure mechanisms was provided by the fractographic SEM analysis (Figure 10, Figure 11, Figure 12 and Figure 13). Representative nanoceramics (CS and LU) generally exhibited smoother fracture surfaces with evidence of filler debonding, particle pull-out, and localized interfacial defects. In contrast, the resin composite blocks (GD and TC) showed rougher fracture surfaces characterized by fracture steps, secondary crack planes, and increased crack-path tortuosity. Similar fracture morphologies have previously been reported in highly filled indirect resin composites and have been associated with more complex crack propagation paths [20].

The fractographic observations suggest that fracture propagation in resin composite blocks involved greater interaction between the advancing crack front and the surrounding microstructure. Crack deflection around filler particles and localized microcracking were frequently observed, whereas interfacial decohesion and filler pull-out appeared more prominent in the nanoceramics. Such differences may be associated with the higher resilience values and, in several cases, the higher reliability observed for the resin composite blocks.

From a classification perspective, resin composite blocks and nanoceramics are already considered distinct subgroups within resin-based CAD/CAM materials [36,39]. The present results support this distinction from a mechanical standpoint, although the absence of consistent statistical differences across all parameters indicates that flexural properties alone are insufficient to fully characterize their behavior. Therefore, additional properties such as fracture toughness, fatigue resistance, and aging behavior should be considered for a more comprehensive assessment of these materials [11,19].

The most relevant finding of this study relates to the evaluation of size-dependent behavior and the validity of Weibull-based scaling. Classical size-effect behavior was clearly observed in the ceramic control, consistent with flaw-controlled fracture mechanisms [20,21,22]. In contrast, resin-based CAD/CAM materials showed limited or inconsistent sensitivity to specimen size, indicating that size-dependent strength cannot be assumed a priori for contemporary resin-based CAD/CAM materials.

The fractographic observations may help explain this behavior. Classical Weibull size-effect theory assumes fracture initiation from a dominant critical flaw within a statistically homogeneous brittle structure. However, the fracture surfaces of the resin-based CAD/CAM materials revealed evidence of crack deflection, microcracking, filler debonding, and crack-path tortuosity, indicating that fracture propagation may be influenced by multiple microstructural mechanisms acting simultaneously. Such mechanisms may reduce the dominance of individual critical flaws and attenuate the size-dependent behavior predicted by classical effective-volume scaling, thereby contributing to the discrepancies observed between predicted and experimental strength values.

Weibull analysis provided a satisfactory statistical description of strength variability for most materials. However, its predictive capacity through effective-volume scaling was limited and strongly material-dependent. Resin-based CAD/CAM materials generally showed an underestimation of strength for larger specimens, whereas the ceramic control exhibited an overestimation, indicating that deviations from classical Weibull scaling are not restricted to resin-based materials.

These findings suggest that discrepancies between predicted and experimental strength cannot be attributed solely to material composition. Instead, they highlight limitations of classical effective-volume scaling when applied under flexural loading conditions, where stress gradients and the relative contribution of surface and volume flaws may differ from the assumptions of the weakest-link theory. Previous studies have shown that Weibull-based approaches are highly sensitive to flaw distribution, effective stressed volume, and testing configuration, and that deviations from ideal Weibull behavior may occur even in conventionally brittle ceramics [20,40,41]. Within this context, the present results indicate that such deviations may also occur under standard flexural testing conditions in resin-based CAD/CAM materials, suggesting that the applicability of classical effective-volume scaling should be evaluated on a material-specific basis rather than assumed a priori.

Although the present results demonstrate that the applicability of classical Weibull effective-volume scaling is material-dependent, the current experimental design was not intended to establish alternative scaling coefficients. Future studies incorporating multiple specimen dimensions and larger datasets should investigate the development of material-specific scaling approaches capable of more accurately describing the mechanical behavior of contemporary resin-based CAD/CAM materials.

This interpretation should nevertheless be made with caution. Although the fractographic analysis provided useful insight into fracture morphology and crack propagation, it remained qualitative and did not include quantitative characterization of flaw populations or critical flaw size. Therefore, the mechanistic interpretation of the observed deviations from classical Weibull behavior should be considered preliminary.

From a clinical perspective, the reduced size sensitivity observed in several resin-based CAD/CAM materials may suggest a lower dependence on critical flaw populations than that of ceramics. However, these findings should not be extrapolated directly to clinical performance, as flexural strength testing does not account for fatigue, aging, or environmental degradation [42,43,44].

The present study has several limitations. Only static flexural testing was performed, and neither fracture toughness nor aging-related phenomena were evaluated. Furthermore, although the sample size was adequate for Weibull analysis, the use of a non-standard specimen width may have influenced stress distribution, despite both geometries behaving as slender beams [45]. Future studies should combine mechanical testing with quantitative fractographic characterization, fracture toughness evaluation, cyclic loading, and aging protocols to better elucidate the mechanisms governing strength reliability and size-dependent behavior in resin-based CAD/CAM materials.

## 5. Conclusions

With the limitations of this study, the following conclusions can be drawn. Resin composite blocks generally exhibited higher flexural strength, elastic modulus, resilience, and reliability than nanoceramics, supporting the existence of distinct mechanical behaviors within resin-based CAD/CAM restorative materials.

Size-dependent strength behavior was strongly material-dependent. Although the lithium disilicate ceramic control exhibited the classical reduction in strength expected with increasing specimen size, most resin-based materials showed limited or inconsistent size dependence. Moreover, no uniform pattern was observed among the resin-based materials evaluated, indicating that specimen geometry and stressed volume influenced mechanical behavior in a material-specific manner.

Agreement between experimentally measured strength values and those predicted by Weibull effective-volume scaling varied considerably among materials. These findings suggest that, although Weibull statistics remain useful for describing strength variability, the direct application of classical effective-volume scaling does not consistently predict the size-dependent behavior of contemporary resin-based CAD/CAM materials, likely reflecting the influence of material-specific microstructural and fracture mechanisms. Consequently, the applicability of Weibull-based strength extrapolation should not be assumed a priori and should be evaluated individually for each material.

## Figures and Tables

**Table 1:** CAD/CAM materials evaluated in the study, including resin-based materials and control groups, with manufacturer, material classification, manufacturer-reported composition, and batch number.

Material	Code	Manufacturer	Class	Composition	Batch No.
Cerasmart 270	CS	GC Corp., Tokyo, Japan.	Nanoceramic	71 wt% barium glass (300 μm), silica (20 nm) + Bis-MEPP, UDMA, DMA, etc.	1907106
Lava Ultimate	LU	3M ESPE, St. Paul, MN, USA.	Nanoceramic	79 wt% silica (20 nm), zirconium particles (4–11 nm), nanoclusters of zirconia/silica (20 nm silica/11 nm zirconia + Bis-GMA, Bis-EMA, TEGDMA, UDMA, etc.).	N868276
Grandio-Block	GD	Voco, Cuxhaven, Germany.	RCB	86 wt% silica, Al-silicate glass (1 μm), pre-polymerised filler (20–40 nm) + UDMA, HEMA, etc.	1902308
Tetric CAD	TC	Ivoclar Vivadent, Schaan, Liechtenstein.	RCB	71.1 wt% silica particles < 20 nm, barium glass < 1 μm + Bis-GMA, Bis-EMA, TEGDMA, UDMA, etc.	W97559
Brava	BR	FGM, Joinville, Brazil.	Nanoceramic	77 wt% vitroceramic filler 40 nm and 5 μm + monomers based on Bis-GMA and other compounds.	230218
Shofu Block	SB	Shofu Inc., Kyoto, Japan.	Nanoceramic	61 wt% silica powder, microfumed silica, zirconium silicate + UDMA, TEGDMA, etc.	0418035
Brilliant Crios	BC	Coltene Holding AG, Alstätten, Switzerland.	RCB	71 wt% barium glass, silica particles + UDMA, Silicic acid, Bis-GMA, Bis-EMA, TEGDMA, etc.	J48662
Katana Avencia	KA	Kuraray Noritake Dental, Tokyo, Japan.	Nanoceramic	71 wt% barium glass, amorphous silica (<1 μm y < 20 nm) + UDMA, TEGDMA (<1 μg/mL), etc.	000397
Estellite P-block	ES	Tokuyama Dental, Tokyo, Japan	Nanoceramic	Silica powder (155–246 nm), silica zirconium particles + UDMA, TEGDMA, etc.	017096
Experimental	EX	Vericom, Gangwon-do, Republic of Korea.	Nanoceramic	80 wt% zirconia, silicate + 20%wt resin.	DL0561A2
G-Cam	GC	Graphenano Nanotechnologies, Valencia, Spain.	PMMA	Polymethylmethacrylate + graphene.	L21021120072
IPS e.Max CAD	EM	Ivoclar Vivadent, Schaan, Liechtenstein.	Ceramic	Lithium disilicate glass-ceramic.	Y24130

**Table 2:** Mean (standard deviation) values of flexural strength, flexural modulus, resilience modulus, and maximum strain for each material and specimen geometry (4 mm and 12 mm).

Mechanical Property	Specimen Geometry	Material											
		CS	LU	GD	TC	BR	SB	BC	KA	ES	EX	GC	EM
Flexural strength (MPa)	4 mm.	161.04 (23.11)	153.13 (17.29)	224.25 (15.73)	185.83 (18.42)	150.92 (17.06)	130.93 (7.99)	198.8 (17.14)	152.16 (18.09)	176.08 (12.92)	166.62 (16.27)	127.11 (9.51)	406.31 (62.50)
	12 mm.	165.91 (25.67)	141.64 (14.06)	208.71 (31.77)	213.36 (8.84)	142.04 (15.68)	135.02 (23.02)	196.17 (26.89)	148.85 (27.11)	164.6 (14.66)	151.39 (11.26)	117.94 (7.53)	243.51 (65.97)
Flexural modulus (GPa)	4 mm.	11.99 (0.60)	14.51 (0.98)	19.95 (1.42)	13.30 (0.724)	13.08 (1.80)	10.15 (0.95)	12.99 (0.72)	9.08 (0.80)	13.41 (0.91)	13.82 (0.84)	3.46 (0.15)	94.09 (7.12)
	12 mm.	12.92 (1.20)	15.15 (0.81)	20.88 (1.35)	13.40 (0.63)	14.00 (0.88)	10.56 (0.73)	13.41 (1.11)	11.51 (0.74)	13.10 (0.80)	13.81 (2.98)	3.63 (0.17)	56.19 (12.67)
Resilience modulus (MPa)	4 mm.	1.10 (0.29)	0.82 (0.17)	1.27 (0.17)	1.31 (0.24)	0.88 (0.15)	0.85 (0.10)	1.53 (0.26)	1.18 (0.23)	1.16 (0.12)	1.01 (0.18)	2.34 (0.33)	0.89 (0.24)
	12 mm.	1.06 (0.33)	0.67 (0.12)	1.07 (0.28)	1.71 (0.17)	0.73 (0.14)	0.88 (0.24)	1.44 (0.31)	0.99 (0.34)	1.04 (0.16)	0.79 (0.10)	1.92 (0.24)	0.54 (0.20)
Maximum strain (%)	4 mm.	1.47 (0.23)	1.16 (0.16)	1.27 (0.13)	1.60 (0.20)	1.34 (0.16)	1.40 (0.13)	1.86 (0.26)	1.69 (0.17)	1.49 (0.10)	1.37 (0.17)	4.39 (0.50)	0.49 (0.06)
	12 mm.	1.48 (0.25)	1.04 (0.11)	1.16 (0.21)	1.96 (0.16)	1.13 (0.11)	1.47 (0.15)	1.81 (0.25)	1.45 (0.27)	1.42 (0.13)	1.24 (0.11)	3.79 (0.36)	0.54 (0.12)

**Table 3:** Comparison of mechanical properties between specimen geometries (4 mm vs. 12 mm) for each material. *p* values obtained from ANOVA are presented for flexural strength, flexural modulus, and resilience modulus. Statistical significance was set at *p* < 0.05.

Material	Flexural Strength	Flexural Modulus	Resilience Modulus
CS_4_ vs. CS_12_	1.000	1.000	1.000
LU_4_ vs. LU_12_	1.000	1.000	0.997
GD_4_ vs. GD_12_	1.000	1.000	0.941
TC_4_ vs. TC_12_	0.743	1.000	0.020
BR_4_ vs. BR_12_	1.000	1.000	0.997
SB_4_ vs. SB_12_	1.000	1.000	1.000
BC_4_ vs. BC_12_	1.000	1.000	1.000
KA_4_ vs. KA_12_	1.000	1.000	0.977
ES_4_ vs. ES_12_	1.000	1.000	1.000
EX_4_ vs. EX_12_	1.000	1.000	0.845
GC_4_ vs. GC_12_	1.000	1.000	0.020
EM_4_ vs. EM_12_	0.000	0.000	0.089

**Table 4:** Characteristic strength, Weibull modulus, and coefficient of determination (R^2^) (**A**) for 4 mm specimens; (**B**) for 12 mm specimens. Values are presented with 95% confidence intervals in parentheses.

**(A)**			
**Material**	**Characteristic** **Strength**	**Weibull** **Modulus**	**R^2^**
CS	170.49 (168.97–172.01)	8.27 (7.53–9.02)	0.979
LU	160.60 (157.66–163.54)	10.26 (9.22–11.31)	0.974
GD	231.39 (207.17–255.62)	16.42 (13.53–19.30)	0.926
TC	194.05 (166.31–221.79)	11.55 (8.41–14.69)	0.839
BR	158.72 (152.50–164.95)	9.71 (8.19–11.23)	0.939
SB	134.46 (118.90–150.02)	19.51 (17.07–21.95)	0.961
BC	206.48 (189.96–223.01)	13.27 (10.86–15.68)	0.921
KA	160.06 (144.95–175.18)	9.69 (7.32–12.05)	0.866
ES	181.77 (176.05–187.49)	16.10 (14.67–17.53)	0.980
EX	175.28 (146.18–204.39)	9.87 (6.62–12.12)	0.780
GC	131.35 (111.62–151.08)	15.65 (12.90–18.40)	0.926
EM	432.11 (421.53–442.68)	7.67 (5.86–9.48)	0.874
**(B)**			
**Material**	**Characteristic Strength**	**Weibull Modulus**	**R^2^**
CS	176.08 (173.89–178.26)	7.88 (6.99–8.77)	0.968
LU	147.89 (138.73–157.05)	11.57 (9.71–13.42)	0.937
GD	224.63 (215.25–234.01)	6.41 (4.60–8.21)	0.829
TC	217.27 (199.96–234.59)	28.90 (26.45–31.35)	0.982
BR	148.81 (138.06–159.55)	10.55 (9.48–10.61)	0.914
SB	145.33 (141.65–149.02)	6.23 (5.54–12.55)	0.913
BC	207.73 (195.41–220.05)	8.39 (6.31–10.48)	0.867
KA	159.96 (158.58–161.35)	6.26 (5.54–6.97)	0.967
ES	170.97 (167.15–174.79)	13.29 (12.11–14.47)	0.980
EX	156.46 (143.59–169.34)	15.37 (13.19–17.55)	0.950
GC	121.94 (87.71–156.16)	17.93 (14.28–21.58)	0.903
EM	267.43 (266.49–268.37)	4.32 (3.75–4.88)	0.958

**Table 5:** Predicted characteristic strength (*σ_θ_*) values for 12 mm specimens calculated from 4 mm specimens using classical Weibull effective-volume scaling, compared with experimentally measured *σ_θ_* values. Prediction error (%) was calculated as the relative difference between predicted and measured *σ_θ_* value.

Material	*σ_θ_* 4 mm (MPa)	*σ_θ_* 12 mm Predicted (MPa)	*σ_θ_* 12 mm Measured (MPa)	Error (%)
CS	170.49	150.17	176.08	−14.72
LU	160.60	144.55	147.89	−2.26
GD	231.39	216.65	224.63	−3.55
TC	194.05	177.11	217.27	−18.48
BR	158.72	142.11	148.81	−4.50
SB	134.46	127.4	145.33	−12.34
BC	206.48	191.21	207.73	−7.95
KA	160.06	142.88	159.96	−10.68
ES	181.77	169.74	170.97	−0.72
EX	175.28	156.93	156.46	+0.30
GC	131.35	122.79	121.94	+0.70
EM	432.11	371.17	267.43	+38.79

## Data Availability

The original contributions presented in this study are included in the article. Further inquiries can be directed to the corresponding author.

## References

[1] Nguyen J.F., Migonney V., Ruse N.D., Sadoun M. (2012). Resin composite blocks via high-pressure high-temperature polymerization. Dent. Mater..

[2] Hussain B., Thieu M.K.L., Johnsen G.F., Reseland J.E., Haugen H.J. (2017). Can CAD/CAM resin blocks be considered as substitute for conventional resins?. Dent. Mater..

[3] Tatiana R., Kharouf N., Matei Dan C., Cournault B., Etienne O. (2026). Who are the Hybrid Ceramics? Bibliometric Review. Int. J. Prosthodont..

[4] Bajraktarova-Valjakova E., Korunoska-Stevkovska V., Kapusevska B., Gigovski N., Bajraktarova-Misevska C., Grozdanov A. (2018). Contemporary dental ceramic materials, a review: Chemical composition, physical and mechanical properties, indications for use. Open Access Maced. J. Med. Sci..

[5] Duarte S., Phark J.H. (2025). Advances in dental restorations: A comprehensive review of machinable and 3D- printed ceramic-reinforced composites. J. Esthet. Restor. Dent..

[6] American Dental Association (2026). CDT: Code on Dental Procedures and Nomenclature.

[7] Gracis S., Thompson V.P., Ferencz J.L., Silva N.R., Bonfante E.A. (2015). A new classification system for all-ceramic and ceramic-like restorative materials. Int. J. Prosthodont..

[8] Rexhepi I., Santilli M., D’Addazio G., Tafuri G., Manciocchi E., Caputi S., Sinjari B. (2023). Clinical applications and mechanical properties of CAD-CAM materials in restorative and prosthetic dentistry: A systematic review. J. Funct. Biomater..

[9] Nguyen J.F., Migonney V., Ruse N.D., Sadoun M. (2013). Properties of experimental urethane dimethacrylate-based dental resin composite blocks obtained via thermo-polymerization under high pressure. Dent. Mater..

[10] Okada K., Kameya T., Ishino H., Hayakawa T. (2014). A novel technique for preparing dental CAD/CAM composite resin blocks using the filler press and monomer infiltration method. Dent. Mater. J..

[11] Ling L., Lai T., Malyala R. (2022). Fracture toughness and brittleness of novel CAD/CAM resin composite block. Dent. Mater..

[12] Coldea A., Swain M.V., Thiel N. (2013). In-vitro strength degradation of dental ceramics and novel PICN material by sharp indentation. J. Mech. Behav. Biomed. Mater..

[13] Fathy H., Hamama H.H., El-Wassefy N., Mahmoud S.H. (2022). Clinical performance of resin-matrix ceramic partial coverage restorations: A systematic review. Clin. Oral Investig..

[14] Wang L., D’Alpino P.H.P., Lopes L.G., Pereira J.C. (2003). Mechanical properties of dental restorative materials: Relative contribution of laboratory tests. J. Appl. Oral Sci..

[15] Ilie N., Hilton T.J., Heintze S.D., Hickel R., Watts D.C., Silikas N., Stansbury J.W., Cadenaro M., Ferracane J.L. (2017). Academy of Dental Materials guidance–Resin composites: Part I–Mechanical properties. Dent. Mater..

[16] (2024). Dentistry—Ceramic Materials.

[17] (2019). Dentistry—Polymer-Based Restorative Materials.

[18] Choi B.J., Yoon S., Im Y.W., Lee J.H., Jung H.J., Lee H.H. (2019). Uniaxial/biaxial flexure strengths and elastic properties of resin-composite block materials for CAD/CAM. Dent. Mater..

[19] Niem T., Youssef N., Wöstmann B. (2019). Energy dissipation capacities of CAD-CAM restorative materials: A comparative evaluation of resilience and toughness. J. Prosthet. Dent..

[20] Quinn J.B., Quinn G.D. (2010). A practical and systematic review of Weibull statistics for reporting strengths of dental materials. Dent. Mater..

[21] Fischer H., Rentzsch W., Marx R. (2002). A modified size effect model for brittle nonmetallic materials. Eng. Fract. Mech..

[22] Griffith A.A. (1921). The phenomena of rupture and flow in solids. Phil. Trans. R. Soc. Lond. A.

[23] Danzer R., Börger A., Supancic P., Ruiz Villanueva M.A. (2003). Ein einfacher Festigkeitsversuch für Scheiben aus spröden Werkstoffen. Mater. Werkst..

[24] Kelly J.R. (1995). Perspectives on strength. Dent. Mater..

[25] Quinn G.D. (2003). Weibull strength scaling for standardized rectangular flexure specimens. J. Am. Ceram. Soc..

[26] Wendler M., Belli R., Petschelt A., Mevec D., Harrer W., Lube T., Danzer R., Lohbauer U. (2017). Chairside CAD/CAM materials. Part 2: Flexural strength testing. Dent. Mater..

[27] Eldafrawy M., Karevan Y., Nguyen J.F., Mainjot A. (2023). Interblock and intrablock homogeneity of CAD-CAM composites mechanical properties. Dent. Mater. J..

[28] Grzebieluch W., Mikulewicz M., Kaczmarek U. (2021). Resin composite materials for chairside CAD/CAM restorations: A comparison of selected mechanical properties. J. Healthc. Eng..

[29] Hampe R., Theelke B., Lümkemann N., Eichberger M., Stawarczyk B. (2019). Fracture toughness analysis of ceramic and resin composite CAD/CAM material. Oper. Dent..

[30] Lauvahutanon S., Takahashi H., Shiozawa M., Iwasaki N., Asakawa Y., Oki M., Finger W.J., Arksornnukit M. (2014). Mechanical properties of composite resin blocks for CAD/CAM. Dent. Mater. J..

[31] Yin R., Kim Y.K., Jang Y.S., Lee J.J., Lee M.H., Bae T.S. (2019). Comparative evaluation of the mechanical properties of CAD/CAM dental blocks. Odontology.

[32] Ducke V.M., Ilie N. (2021). Aging behavior of high-translucent CAD/CAM resin-based composite blocks. J. Mech. Behav. Biomed. Mater..

[33] Mokhtar M.M., Farahat D.S., Eldars W., Osman M.F. (2022). Physico-mechanical properties and bacterial adhesion of resin composite CAD/CAM blocks: An in-vitro study. J. Clin. Exp. Dent..

[34] Vichi A., Goracci C., Carrabba M., Tozzi G., Louca C. (2020). Flexural resistance of CAD-CAM blocks. Part 3: Polymer-based restorative materials for permanent restorations. Am. J. Dent..

[35] Mainjot A.K., Dupont N.M., Oudkerk J.C., Dewael T.Y., Sadoun M.J. (2016). From artisanal to CAD-CAM blocks: State of the art of indirect composites. J. Dent. Res..

[36] Marchesi G., Camurri Piloni A., Nicolin V., Turco G., Di Lenarda R. (2021). Chairside CAD/CAM materials: Current trends of clinical uses. Biology.

[37] Baba T., Nakashima Y., Takahashi S., Matsubara T., Yin L., Nakanishi Y. (2019). Micro-slurry jet for surface processing of dental ceramics. Biosurf. Biotribol..

[38] Ling L., Ma Y., Malyala R. (2021). A novel CAD/CAM resin composite block with high mechanical properties. Dent. Mater..

[39] Sulaiman T.A. (2020). Materials in digital dentistry–A review. J. Esthet. Restor. Dent..

[40] Belli R., Lohbauer U. (2021). The breakdown of the Weibull behavior in dental zirconias. J. Am. Ceram. Soc..

[41] Danzer R., Supancic P., Pascual J., Lube T. (2007). Fracture statistics of ceramics–Weibull statistics and deviations from Weibull statistics. Eng. Fract. Mech..

[42] Kelly J.R. (2004). Dental ceramics: Current thinking and trends. Dent. Clin. N. Am..

[43] Zhang Y., Sailer I., Lawn B.R. (2013). Fatigue of dental ceramics. J. Dent..

[44] Belli R., Wendler M., de Ligny D., Cicconi M.R., Petschelt A., Peterlik H., Lohbauer U. (2017). Chairside CAD/CAM materials. Part 1: Measurement of elastic constants and microstructural characterization. Dent. Mater..

[45] Papathanasiou I., Kamposiora P., Dimitriadis K., Papavasiliou G., Zinelis S. (2023). In vitro evaluation of CAD/CAM composite materials. J. Dent..

